# Multicenter validation of cancer gene panel-based next-generation sequencing for translational research and molecular diagnostics

**DOI:** 10.1007/s00428-017-2288-7

**Published:** 2018-01-27

**Authors:** B. Hirsch, V. Endris, S. Lassmann, W. Weichert, N. Pfarr, P. Schirmacher, V. Kovaleva, M. Werner, I. Bonzheim, F. Fend, J. Sperveslage, K. Kaulich, A. Zacher, G. Reifenberger, K. Köhrer, S. Stepanow, S. Lerke, T. Mayr, D. E. Aust, G. Baretton, S. Weidner, A. Jung, T. Kirchner, M. L. Hansmann, L. Burbat, E. von der Wall, M. Dietel, M. Hummel

**Affiliations:** 1 Campus Mitte, Institute of Pathology, Charité-University Medicine Berlin, Virchowweg 15, 10117 Berlin, Germany; 2 German Cancer Consortium (DKTK) Partner Site, and German Cancer Research Center (DKFZ), 69120 Heidelberg, Germany; 3 Institute of Pathology, University Medicine Heidelberg, Im Neuenheimer Feld 224, 69120 Heidelberg, Germany; 4 Institute for Surgical Pathology, Medical Center, Faculty of Medicine, University of Freiburg, Breisacherstraße 115A, 79106 Freiburg, Germany; 5 Institute of Pathology, Technical University Munich (TUM), Munich, Germany; 6 Institute of Pathology and Neuropathology, University Hospital Tuebingen, Eberhard-Karls-University, Liebermeisterstraße 8, 72076 Tuebingen, Germany; 7 Department of Neuropathology, Heinrich Heine University Duesseldorf and Biological and Medical Research Center (BMFZ), Genomics and Transcriptomics Laboratory, Heinrich Heine University Duesseldorf, 40225 Duesseldorf, Germany; 8 Institute of Pathology, University Hospital Carl Gustav Carus, Fetscherstraße 74, 01307 Dresden, Germany; 9 Institute of Pathology, Ludwig-Maximilians University Munich, Thalkirchner Straße 36, 80337 Munich, Germany; 10 Dr. Senckenberg Institute of Pathology, University Hospital, Goethe-University, Theodor-Stern-Kai 7, 60590 Frankfurt am Main, Germany

**Keywords:** German Cancer Research Centers (DKTK-sites), Multicenter trial, FFPE cancer samples, Amplicon-based next-generation sequencing, PGM™, MiSeq™

## Abstract

The simultaneous detection of multiple somatic mutations in the context of molecular diagnostics of cancer is frequently performed by means of amplicon-based targeted next-generation sequencing (NGS). However, only few studies are available comparing multicenter testing of different NGS platforms and gene panels. Therefore, seven partner sites of the German Cancer Consortium (DKTK) performed a multicenter interlaboratory trial for targeted NGS using the same formalin-fixed, paraffin-embedded (FFPE) specimen of molecularly pre-characterized tumors (*n* = 15; each *n* = 5 cases of Breast, Lung, and Colon carcinoma) and a colorectal cancer cell line DNA dilution series. Detailed information regarding pre-characterized mutations was not disclosed to the partners. Commercially available and custom-designed cancer gene panels were used for library preparation and subsequent sequencing on several devices of two NGS different platforms. For every case, centrally extracted DNA and FFPE tissue sections for local processing were delivered to each partner site to be sequenced with the commercial gene panel and local bioinformatics. For cancer-specific panel-based sequencing, only centrally extracted DNA was analyzed at seven sequencing sites. Subsequently, local data were compiled and bioinformatics was performed centrally. We were able to demonstrate that all pre-characterized mutations were re-identified correctly, irrespective of NGS platform or gene panel used. However, locally processed FFPE tissue sections disclosed that the DNA extraction method can affect the detection of mutations with a trend in favor of magnetic bead-based DNA extraction methods. In conclusion, targeted NGS is a very robust method for simultaneous detection of various mutations in FFPE tissue specimens if certain pre-analytical conditions are carefully considered.

**Electronic supplementary material:**

The online version of this article (10.1007/s00428-017-2288-7) contains supplementary material, which is available to authorized users.

## Introduction

Whole genome sequencing (WGS) or whole exome sequencing (WES) are excellent tools for the comprehensive and explorative detection of genetic alterations in tumor DNA extracted from cancer cells and tissues [10]. However, these techniques produce overwhelming amounts of data that require extensive bioinformatics analyses [5]. Moreover, for proper interpretation of disease-associated somatic variations in the tumor DNA, sequencing of constitutive germline specimen from the same patient is essential [8]. In addition, high variability in mutation call rates and limited concordance among analysis pipelines have been reported in a comparative analysis of WGS data within the International Cancer Genome Consortium [1]. Therefore, also in terms of high costs and long processing times, WGS or WES are currently not widely used for timely molecular routine diagnostics of cancer tissues and associated therapeutic stratification of tumor patients. For the diagnostic detection of cancer-associated somatic alterations relevant for therapeutic decisions, targeted amplicon-based Next-Generation Sequencing (NGS) of cancer gene panels has evolved as a promising approach [19]. For this method, short genomic regions are captured or PCR-amplified at the initial part of library preparation, and subsequently subjected to NGS. The amount of data created and bioinformatics are limited, while sequencing depth can be very high, thus allowing for sensitive detection of even subclonal mutations [18, 29].

The two main NGS platforms available at the time of our multicenter study for targeted parallel sequencing were the Ion Torrent Personal Genome Machine (PGM™) from Thermo Fisher Scientific [9, 22] and the MiSeq™ desktop sequencer from Illumina [23]. There is only limited data to address the question whether NGS performed independently on both platforms provide the same results across different laboratories [7, 16]. Since gene panel NGS is becoming more and more popular in molecular diagnostics of cancer and many clinical trials are carried out in a multicenter setting, the assessment of interlaboratory reliability of this method is of uttermost importance.

Therefore, we performed a multicenter trial within the German Cancer Consortium (DKTK) to evaluate the comparability of different pre-analytic workflows and both sequencing platforms for diagnostic targeted NGS.

Our joint data sets from different sequencing sites and cross-validation clearly identify targeted NGS as a valuable and valid approach independent of the NGS library preparation and the sequencing platform (PGM™ or MiSeq™) used. However, local processing of FFPE sections involving microdissection of selected tumor areas and different DNA extraction protocols demonstrated—particularly in case of low DNA quality—that allelic frequencies may vary or that mutations remain undetectable in a few cases. Thus, targeted NGS can be applied reliably in multicenter studies, assuming careful selection and validation of the DNA extraction method used.

## Material and methods

### Case selection and tissue preparation

A total of seven laboratories contributed to this multicenter trial, including six pathology laboratories and one neuropathology laboratory (Berlin (B), Dresden (DD), Freiburg (FR), Heidelberg (HD), Munich (M), Tuebingen (TUE), and Düsseldorf (D)). M provided lung cancer samples but did not perform sequencing analyses. PGM analyses were performed in B, HD, and D; MiSeq™-based analyses were carried out in DD, FR, and TUE and in addition to PGM™-based sequencing in B. For the generation of NGS data, centrally extracted DNA as well as unstained FFPE sections for local processing of 15 cancer cases (five Breast, five Lung, and five Colorectal carcinomas) were distributed to each partner site. Selection of cases was based on mutations previously ascertained by conventional sequencing analyses including mutations in *KRAS* detected in each of the five Colon cancer samples. In Breast cancer samples #1, #3, #4, and #5, *PIK3CA* mutations and a *PTEN* mutation in Breast cancer sample #2 were pre-analyzed. Mutations in *EGFR* were ascertained previously in all five Lung cancer samples. Furthermore, the histopathology of each FFPE cancer specimen was reviewed by expert pathologists to confirm diagnosis and determine the tumor cell content.

For local processing, FFPE tissue sections provided were reviewed by pathologists at the respective partner site and tumor areas were indicated for microdissection. DNA was extracted from microdissected tumor areas using either Maxwell® Rapid Sample Concentrator (RSC) Instrument and the Maxwell® 16 FFPE plus LEV DNA Purification Kit (Promega GmbH, Mannheim, Germany), High Pure FFPE DNA Isolation Kit (Roche Diagnostics, Mannheim, Germany), or QIAamp DNA FFPE Tissue Kit (QIAGEN, Hilden, Germany). DNA was quantified by Qubit dsDNA HS assay kit (QIAGEN), the NanoDrop instrument (Thermo Fisher Scientific, Dreieich, Germany), or the TaqMan® RNase P Detection Reagents Kit (Thermo Fisher Scientific). DNA yield and sequencing results may therefore also in part vary between sites due to the tumor areas selected for downstream processing and analysis as well as regarding the thresholds of the DNA quantification protocols.

### Sensitivity testing

To exemplarily test for sensitivity, DNA extracted from FFPE tonsillar tissue was mixed with different proportions of DNA (50, 10, 3, and 1%) derived from formalin-fixed and paraffin-embedded cells of the Colon cancer cell line LoVo (CCL-229, obtained from ATCC) which was processed according to the same protocols as used for the cancer tissue samples. This cell line carries mutations in the genes for *KRAS* (G13D), and *FBXW7* (R387C), and a polymorphism in *CKIT* (M541L). In addition, a gene amplification is present in the chromosomal region where *KRAS* is located [3]. Centrally extracted DNA was distributed to the participating sequencing partner sites.

### Study design—library preparation and sequencing

**Fig. 1:**
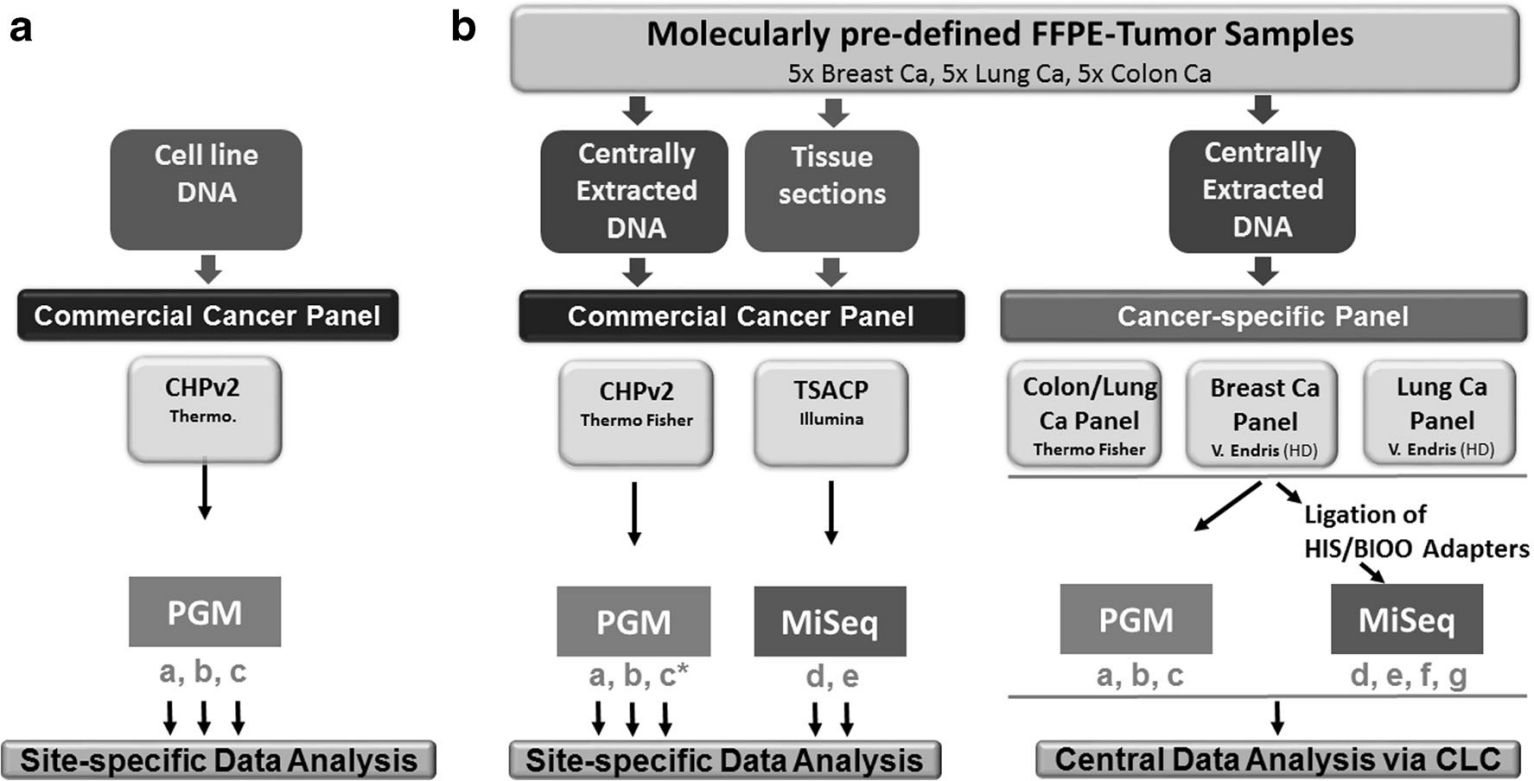
Multicenter study design for targeted NGS. **a** A commercial gene panel (Cancer Hotspot panel 2, CHPv2, Thermo Fisher Scientific) was applied to DNA from the LoVo cell line at four distinct dilutions at three PGM™ sequencing sites (a, b, and c) to demonstrate exemplarily sensitivity. Bioinformatics was performed locally. **b** Genomic DNA from 15 molecularly pre-characterized tumor samples (five breast, five lung, and five colon cancer cases) was analyzed with commercially available and custom-designed cancer gene panels on PGM™ and MiSeq™ benchtop sequencers at seven sequencing sites (a, b, c, d, e, f, and g). FFPE tissue sections of the very same tumor samples were delivered to the sites a, b, d, and e for local microdissection, DNA Isolation, QC/quantification, and commercial panel sequencing. Partner site c did not receive tissue sections for local DNA extraction and applied only centrally extracted DNA to commercial (c*) and cancer-specific gene panel sequencing (c). Bioinformatics of commercial cancer panel-based data was performed individually whereas cancer-specific panel-based data were collected centrally and compiled

Commercial cancer gene panel, Ion AmpliSeq™ Cancer Hotspot Panel v2 CHPv2 [28] (Thermo Fisher Scientific), consisting of 207 amplicons covering hotspot mutations of 50 genes, and TruSeq® Amplicon Cancer Panel (TSACP, Illumina) [12, 24], consisting of 212 amplicons of 48 genes, were employed at three PGM and two MiSeq benchtop sequencing platforms. Commercial cancer gene panels were applied to analyze centrally extracted DNA from 15 pre-characterized tumor samples as well as site-specific/locally isolated DNA from the very same FFPE tumor specimens (exception being site c) that have been distributed. Bioinformatics was initially performed at each sequencing site individually (left part of Fig. 1b).

In addition, tumor entity-specific, custom-designed gene panels (Lung/NSCLC cancer and Breast cancer panels) and a commercially available “community panel” (Colon/Lung cancer panel, (CL2), Thermo Fisher Scientific) were applied at seven partner sites for sequencing the centrally isolated and distributed DNA of 15 tumor samples. The custom-designed Lung and Breast cancer panels cover 22.78 kb/22.46 kb and consist of 139/136 primer pairs, respectively. Both custom panels were designed by V. Endris (HD) [13] and manufactured as single primer pools by Thermo Fisher Scientific. Tumor entity-specific panels were used for library preparation and subsequent sequencing at three PGM sequencing sites. To allow sequencing of tumor entity-specific panel-based libraries on MiSeq benchtop sequencers, libraries were prepared centrally using NEXTflex™ Rapid DNA-Seq Kit in combination with NEXTflex® Barcode-Kit (HIS/BIOO Scientific Corporation, Austin, TX, USA) and distributed to four MiSeq™ sites, i.e., two additional sites were implemented. Therefore, tumor entity-specific panel-based sequencing was performed at seven different laboratories from the same library samples, whereas bioinformatics was performed at a single partner site employing “CLC-Genomics Workbench”, Version 1.5.2 (Qiagen).

### Specification of amplicon gene panels

#### TruSeq® amplicon cancer panel (TSACP)

The commercially available TSACP (Illumina) is able to detect mutational hotspots of 48 cancer-related genes, consisting of 212 pairs of primers designed to bind at the flanking genomic regions of interest covering 35 kb. Two hundred fifty nanograms of input gDNA were used for preparation of PCR-free libraries. Sequencing was performed using MiSeq™ benchtop sequencer according to the manufacturer’s instructions.

#### Ion AmpliSeq™ cancer hotspot panel v2 (CHPv2)

The CHPv2 is provided by Thermo Fisher Scientific as a single primer pool which covers hotspots and targeted regions of 50 cancer genes consisting of ~ 2800 COSMIC mutations. The CHPv2 consists of 207 primer pairs enabling a multiplex PCR approach to target genomic areas of interest, spanning 31 kb in total. Ten nanograms gDNA was applied for library preparation. Sequencing was performed using the PGM™ benchtop sequencer according to the manufacturer’s instructions.

#### Ion AmpliSeq™ Colon Lung cancer panel v2 (CL2)

The CL2 is a gene panel designed for the analysis of 22 genes relevant for Colon and Lung cancer. It is available as a single primer pool from Thermo Fisher Scientific. The CL2 was developed by the members of the OncoNetwork Consortium in cooperation with Thermo Fisher Scientific and consists of 92 primer pairs [27]. Library preparation and sequencing using the Ion Torrent PGM™ platform was performed according to the manufacturer’s instructions.

#### Custom panels for Breast cancer (IAD35185) and Lung cancer (IAD34679)

Both custom panels were designed by V. Endris and manufactured as single primer pools by Thermo Fisher Scientific. The custom Breast and Lung cancer panels cover 22.46 and 22.78 kb, and consist of 136 [13] and 139 primer pairs, respectively. Library preparation and sequencing using the Ion Torrent PGM™ platform was performed according to the manufacturer’s instructions.

#### HISS/BIOO-adapter ligation

In order to make Thermo Fisher Scientific amplicon panels compatible for the Illumina technology, NEXTflex™ Rapid DNA-Seq Kit in combination with NEXTflex® Barcode-Kit (HIS/BIOO Scientific Corporation, Austin, TX, USA) [15] was applied according to the manufacturer’s instructions. Proper adapter ligation was confirmed by Bioanalyzer High Sensitivity DNA Assay (Agilent, Santa Clara, CA, USA), based on the different length of successfully ligated amplicons.

### Data analysis

**Fig. 2:**
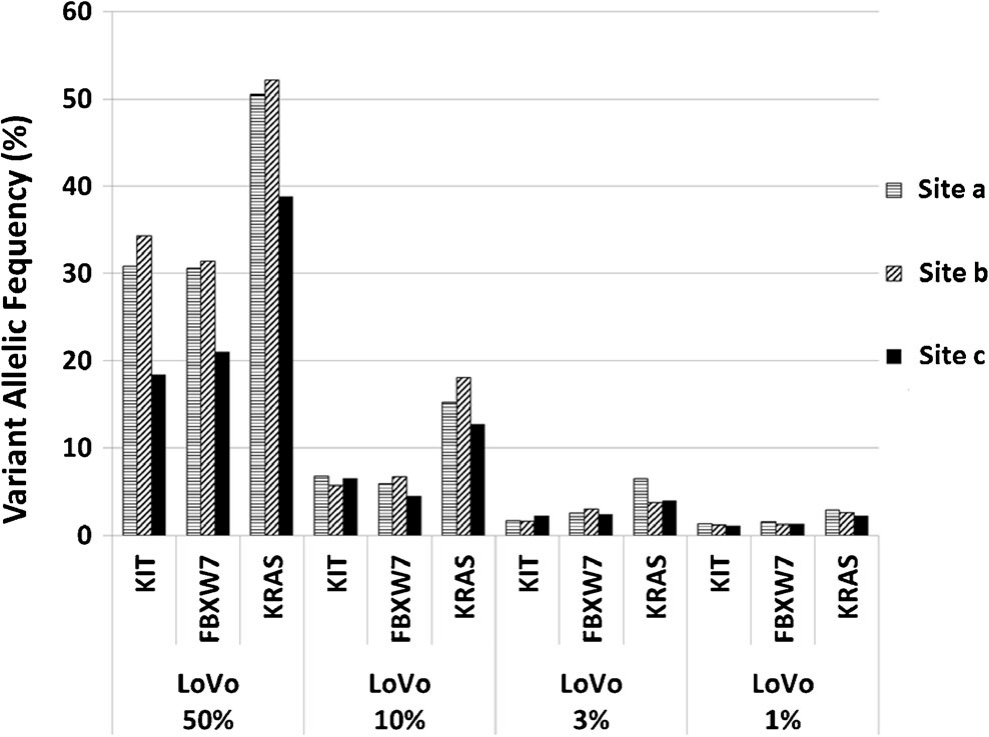
Sensitivity and reproducibility. Four DNA dilutions of the cell line LoVo were prepared, ranging from 50, 10, 3, to 1% on the background of un-mutated human tonsil DNA. Cancer Hotspot Panel v2 (CHPv2, Thermo Fisher Scientific) was applied at three PGM sequencing sites (a, b, c). LoVo shows variants of *KRAS* (G13D and genomic gene amplification), *CKIT* (M541L), and *FBXW7* (R505C). Detected allelic frequencies (%) are illustrated

PGM™ data were analyzed by the Torrent Suite Software (v4.0 or higher, Thermo Fisher Scientific). After alignment to the hg19 human reference genome, the Variant Caller (Thermo Fisher Scientific) was applied to filter polymorphic variants using the CHPv2.20131001-bed-file, Colon- and Lung- (ColonLungV2.20140523.designed), or custom-made Breast or Lung cancer hotspot bed files (Breast Cancer Panel, IAD35185, Lung Cancer Panel, IAD34679, Thermo Fisher Scientific). Nucleotide variations with less than 5% allelic variant frequency were masked, except for the sensitivity testing in order to display low frequency variants (Fig. 2). All detected variants were manually reviewed by the use of the Integrative Genomics Viewer (IGV V.2.1, Broad Institute, Cambridge, MA, USA) [6, 21, 26]. Illumina-based data evaluation, annotation, prediction of significance of variants, and read depths across all positions/coverage was conducted by the use of VariantStudio v2.2 (Illumina). Bioinformatics of commercial cancer panel-based data was performed individually. Bioinformatics of cancer-specific panel-based data was executed centrally by the use of “CLC-Genomics Workbench” Version 1.5.2 (Qiagen).

## Results

### Design for multicenter testing

This multicenter interlaboratory trial for amplicon-based gene panel NGS of 15 molecularly pre-characterized cancer tissue samples and DNA extracts from Breast, Lung, and Colon cancer cases (five cases, each) and four different dilutions of DNA from the Colon cancer cell line LoVo was carried out by seven DKTK partner sites as described above (Fig. 1 and Material and Methods). Two different NGS platforms and several cancer gene panels (commercially available and custom designed) were included to analyze centrally extracted DNA as well as locally processed tissue samples of the same cases.

### Sensitivity and reproducibility

Dilutions (50, 10, 3, and 1%) of DNA extracted from FFPE cell line blocks of the colon cancer cell line LoVo in DNA derived from a FFPE tonsil were used to exemplarily determine the sensitivity and reproducibility of the commercially available amplicon panel CHPv2. DNA-aliquots of the four dilutions were provided for NGS to the three PGM sites. All three participants detected the expected mutations, i.e., *KRAS* (G13D), and *FBXW7* (R505C) and polymorphism *CKIT* (M541L), present in LoVo cells at each dilution. Results of the three PGM™ sequencing sites for the listed variant frequencies are depicted in Fig. 2 and supplementary Table 1. Data reflect the expected allelic frequencies with good concordance. Of note, we discovered a *KRAS* gene amplification (four copies) in LoVo by SNP array analysis (data not shown). This explains allelic frequency twofold higher than expected (Fig. 2, supplementary Table 1). The coverage of all sequenced targets always reached > 1000× at all sites for each sample.

### Commercial cancer gene panel-based NGS analysis of the cancer tissue samples

Commercial cancer panel-based NGS was analyzed initially locally, i.e., individual bioinformatics pipelines were applied. Results from centrally extracted DNAs were most homogeneous, irrespective of the partner site, NGS technology (PGM™ or MiSeq™), and bioinformatics pipeline involved. DNA analysis of locally processed tissue sections provided less homogeneous results, especially between different NGS platforms. Sequencing only with centrally provided DNA was performed at site c for all cases, at site e for ColonCa#3, ColonCa#4, and ColonCa#5 and at site e for BreastCa#4.

**Fig. 3:**
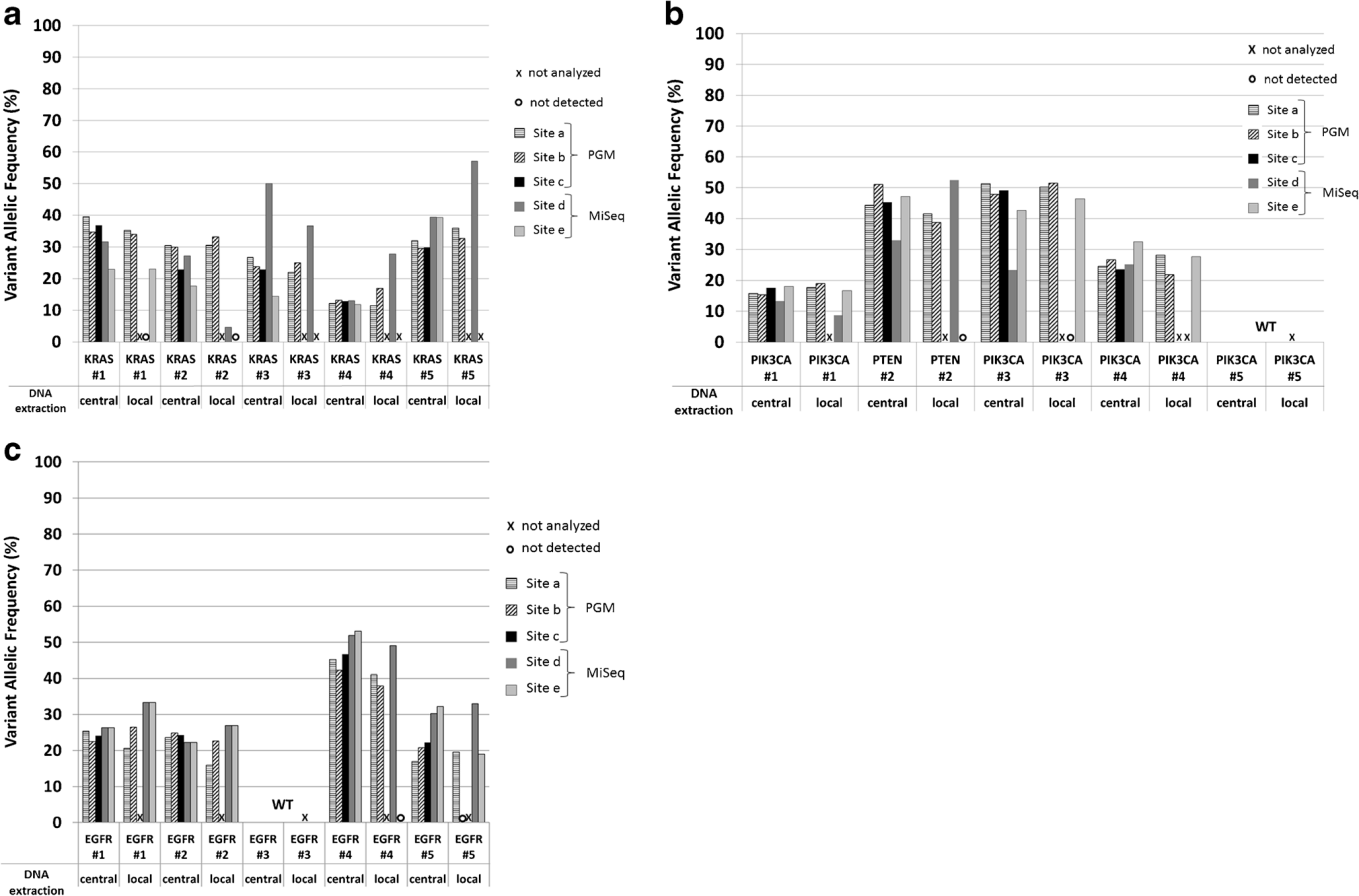
Analysis of 15 FFPE cancer samples with commercial cancer panels. Centrally as well as locally extracted DNA of molecularly pre-characterized cancer samples was sequenced by commercial cancer panels (CHPv2 and TSACP) at five different sequencing sites. Mutations ascertained by conventional Sanger or pyro-sequencing and reproduced by NGS are listed in supplementary Table 2. a Analysis of five Colon cancer samples. **b** Analysis of five Breast cancer samples. **c** Analysis of five Lung cancer samples, (#1–#5, respectively). Variant allelic frequencies, detected at different partner sites, are illustrated by bars as indicated. Samples not analyzed are indicated by “X”; variants not detected are indicated by open circles “**○**.” *WT* wild type; a, b, and c PGM™ sequencing sites; d and e MiSeq™ sequencing sites

All pre-characterized mutations were detected in the Colon cancer (Fig. 3a), Breast cancer (Fig. 3b), and Lung cancer (Fig. 3c) samples at each sequencing site whenever centrally extracted DNA was used for NGS. Exact variant frequencies for all cancer entities, both types of DNA (centrally and locally extracted) and all sequencing sites are listed in supplementary Table 2. The methods used for local DNA extraction represent a considerable variety, which is also reflected in the amount of extracted DNA (supplementary Table 5). Since centrally extracted DNA enabled the detection of all pre-characterized mutations and since all other variables remained consistent in the NGS experiments (Fig. 3a–c), we identified local processing including DNA extraction as the most critical factor for comparable NGS data.

Whereas centrally extracted DNA enabled highly consistent detection of all variants irrespective of the NGS method applied, the use of locally processed and extracted DNA from the Colon cancer samples prevented the detection of some variants in two samples: ColonCa#1, *KRAS* mutation (G13D) at sequencing site d, and ColonCa#2, *KRAS* mutation (G12V) at sequencing site e.

Detected allelic frequencies of pre-characterized variants by the different NGS sequencing sites are listed in supplementary Table 2. Variants undetectable in locally extracted DNA of breast cancer samples were *PTEN* mutation (YQ/*) of BreastCa#2 at sequencing site e and PIK3CA mutation (E545K) in BreastCa#3 at sequencing site d (Fig. 3b).

Commercial CHPv2- and TSACP-based NGS of locally extracted Lung cancer DNA samples revealed that for LungCa#4, *EGFR* deletion (T698fs) was not detected at sequencing site e, and *EGFR* deletion (L694_T698del) of LungCa#5 was not identified at sequencing site b (Fig. 3c, supplementary Table 2).

### Cancer-specific panel-based NGS of cancer samples

**Fig. 4:**
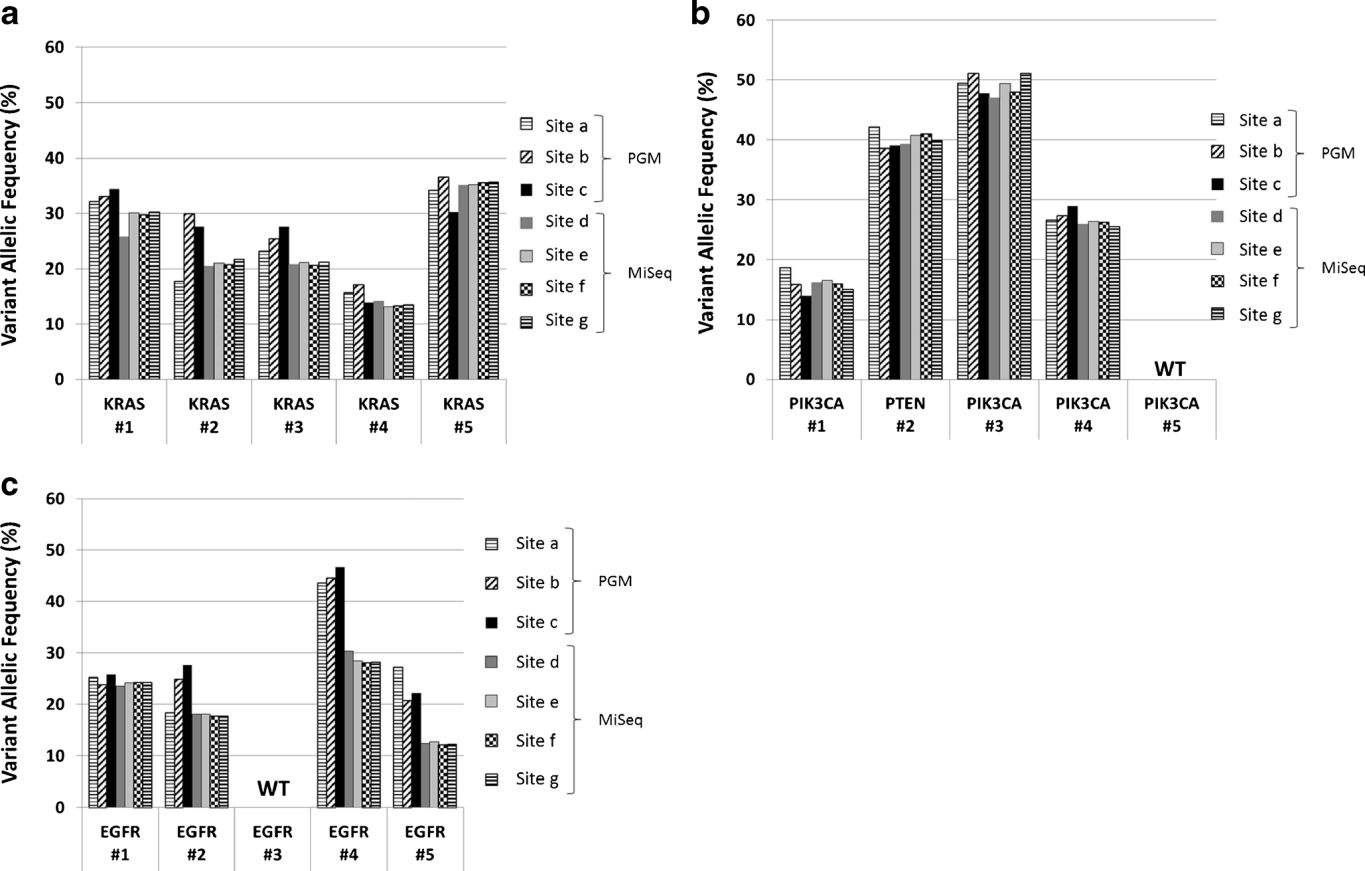
Disease-specific gene panel-based analysis of FFPE cancer samples. Centrally extracted DNA of molecularly pre-characterized cancer samples was sequenced using cancer-specific, custom-designed cancer panel at seven sequencing sites. The same cases as depicted in Fig. 3 are used. **a** Colon/Lung Ca panel-based analysis of five Colon cancer samples. **b** Custom-designed Breast cancer panel-based analysis of five Breast cancer samples. **c** Custom-designed Lung Cancer panel-based analysis of five Lung cancer samples (#1–#5, respectively). Mutations ascertained by conventional Sanger or pyro-sequencing and reproduced by NGS are listed in supplementary Table 2. Detected variant allelic frequencies of *KRAS* (Colon Ca), *PIK3CA*/*PTEN* (Breast Ca), and *EGFR* mutations (Lung Ca) are illustrated by bars in corresponding colors/patterns (a, b, and c represent PGM; d, e, f, and g represent MiSeq sequencing sites). *WT* wild type

Disease-specific amplicon panels specifically designed for variants frequently present in Colon cancer (Fig. 4a), Breast cancer (Fig. 4b), and Lung cancer patients (Fig. 4c) were additionally applied at all 15 cases. For this task, only centrally extracted DNA samples were sequenced at three PGM™ and four MiSeq™ platforms. Since all disease-specific gene panels were designed for IonTorrent technology, library preparation was performed at PGM™ sites (a, b, c) according to the manufacturer’s instructions. In order to render amplicon panel compatible to MiSeq™ analysis (d, e, f, g), NEXTflex™ Rapid DNA-Seq Kit in combination with NEXTflex® Barcode-Kit was carried out centrally according to the manufacturer’s instructions and these libraries were distributed to MiSeq™ sites for sequencing. Bioinformatics of cancer-specific gene panel-based analysis was not performed locally, like commercial cancer gene panel-based NGS, but centrally via “CLC-Genomics Workbench” and achieved a very high degree of uniformity of results, i.e., all pre-characterized variants in the colon (Fig. 4a), breast (Fig. 4b), and Lung cancer samples (Fig. 4c) were detected at each sequencing site. The performance of cancer-specific panels was equally good with Ion Torrent PGM™ and MiSeq™ sequencing machines.

### Additional mutations

In addition to the confirmation of pre-characterized mutations (Figs. 3 and 4), additional variants were consistently detectable by commercial amplicon panel-based NGS, including variants in *TP53*, *APC*, *ATM*, *PIK3CA*, and *JAK3*. Frequencies for all additionally detected variants at all sequencing sites using the commercial cancer panels are listed in supplementary Table 4.

## Discussion

This study addresses an intensively discussed but still open question regarding the comparability of different NGS platforms and gene panels for the detection of clinically relevant mutations performed across different centers. To clarify the feasibility of this multicenter concept, seven molecular pathology laboratories of the German Cancer Consortium (DKTK) joined forces and employed different gene panels and two NGS platforms (3 × PGM™; 4 × MiSeq™) for a multicenter trial of 15 pre-characterized cancer cases and an exemplary series of a cell line DNA dilution. The results obtained were highly comparable if certain preconditions were considered.

Next-generation sequencing (NGS) is not a one single approach, but reflects a very broad range of applications ranging from the sequencing of a limited number of small amplicons with high coverage to whole genome sequencing [14]. However, even within each application, there are many different technical and methodological variants. Concerning the use of targeted amplicon-based NGS for the detection of mutations in cancer samples, major variables are tumor cell content, DNA quality, individual gene panels, and last but not least the NGS platform used. In our multicenter trial, we covered all of these aspects.

To identify the most critical steps for amplicon-based NGS and to establish guidelines along with standardized procedures [4], we provided not only centrally extracted DNA, but also tissue sections for local processing, including DNA extraction. While the use of centrally extracted DNA resulted in very homogeneous NGS data across various gene panels and NGS platforms, data generated with locally selected tumor areas and extracted DNA are more variable. Differences in the microdissection, performed for enrichment of tumor cells, appears to be of minor impact since allelic frequencies of somatic mutations detected demonstrated a narrow range across different laboratories. Since all other variables kept constant, the type of DNA extraction appears to be one of the major sources of variability. However, careful re-inspection of the various DNA extraction methods used at the partner sites revealed no clear-cut preference and there is only tendency in favor of magnetic bead-based technology that was also applied for central DNA extraction.

To address potential additional effects by different gene panels to allelic frequencies of the detected somatic mutations, we investigated the same cancer samples employing various gene panels with overlapping genes in our multicenter setting. To this end, we employed commercial cancer gene panels as well as customized panels for specific cancer types. Despite the fact of using these different gene panels, the overall performance for the overlapping gene regions was very similar. As depicted in Fig. 4, analyses of the same batch of centrally extracted and distributed DNA samples using tumor-specific gene panels at seven different sequencing sites revealed homogeneous results (supplementary Table 3).

Another topic of frequent discussion arises from the question which of the two leading benchtop sequencing instruments (Ion Torrent PGM™ [9, 22] and Illumina MiSeq™) [23] might be better suited for targeted NGS, especially if DNA is derived from FFPE tissue specimens. This question was also covered by our multicenter trial by processing and sequencing the same molecularly pre-characterized 15 cancer cases using both types of instruments at the participating NGS centers. Irrespective of instrument-specific differences, pre-characterized mutations were identified reliably on both NGS platforms at all partner sites when centrally extracted DNA was analyzed. This holds also true for additionally detected variants of certain genes such as *APC*, *MET*, *TP53*, *PIK3CA*, and others not previously characterized by conventional sequencing technologies in our case series.

Precision medicine, i.e., stratification of patients for targeted treatment on the basis of specific molecular alterations detected in the tumor tissue, is of rapidly growing importance in daily clinical practice [17]. Due to the increasing demand for the related diagnostic NGS tests, the comparability, reproducibility, and reliability of diagnostic findings is of fundamental relevance for targeted treatment and translational research especially in a multicenter situation or for diagnostic data from various sites. Therefore, interlaboratory trials comparing the entire workflow for targeted NGS including tissue processing (microdissection), DNA extraction, DNA quantification [25], different gene panels [11], and different NGS platforms [20] on the basis of identical pre-characterized routine tissue specimens at different sequencing sites with different NGS machines of the same and different vendors are important and should be established as a regular measure of external quality assessment [2].

In conclusion, our results of the DKTK multicenter comparison of targeted NGS indicate that this approach is in general a robust and reproducible technique that generates reliable sequencing data within a short period of time. The data generated across different NGS sites using different gene panels and different NGS platforms are very similar. However, the choice of DNA extraction method and poor quality of DNA might impact the reliable identification of mutations. To overcome this potential obstacle, careful validation of the DNA extraction procedure is strongly recommended not only for multicenter activities but also to ensure reliable diagnostic information locally. In conclusion, targeted NGS can be applied by qualified laboratories for clinical studies and routine diagnostics employing validated gene panels and appropriate DNA extraction methods to establish and maintain a reliable workflow from tissue sample to NGS data with diagnostic and therapeutic consequence.

## Electronic supplementary material

Table S1 *KRAS* (G13D), *CKIT* (M541 L), and *FBXW7* (R505C) variant allelic frequencies (%) of cell line DNA dilutions (LoVo), detected at the three PGM™ sequencing sites (a, b, c) that applied the commercial cancer amplicon panel (CHPv2). (DOCX 263 kb)

Table S2 Variant allelic frequencies (%) of analyzed tumor DNAs derived from all sequencing sites (PGM™: a, b, and c; MiSeq™: d and e) that applied commercial broad cancer panels (CHPv2 or TSACP) for NGS. Samples not analyzed are indicated by “X”. Variants not detected are indicated by open circles “**○**”. WT = wild type. Local or central DNA extraction is indicated. (DOCX 257 kb)

Table S3 Detected variant allelic frequencies (%), using centrally extracted tumor DNAs and cancer-specific gene panels at all sequencing sites (PGM™: a, b, c; MiSeq™: d, e, f, and g). Mean value (AVG) and standard deviation (STDN) are given. WT = wild type. (DOCX 266 kb)

Table S4 Additional variants consistently detected at all sequencing sites using the commercial cancer gene panel-based NGS approach. Allelic variant frequencies are indicated (%). Sequencing site “c” did not perform local DNA extractions. (DOCX 266 kb)

Table S5 Methods used for local DNA extraction of FFPE patient samples at four sequencing sites. DNA concentrations (ng/μl) and total volume (μl) of individual samples are shown. Samples in which known mutations have not been detected by targeted NGS are highlighted (filled gray). (DOCX 46 kb)
