# Supplementary material for: Multicenter validation of cancer gene panel-based next-generation sequencing for translational research and molecular diagnostics
Source: Virchows Arch. 2018 Jan 27;472(4):557–65. doi: 10.1007/s00428-017-2288-7 (PMC5924673; doi:10.1007/s00428-017-2288-7)
Supplement: Supplementary file 1 — KRAS (G13D), CKIT (M541 L), and FBXW7 (R505C) variant allelic frequencies (%) of cell line DNA dilutions (LoVo), detected at the three PGM™ sequencing sites (a, b, c) that applied the commercial cancer amplicon panel (CHPv2). (DOCX 263 kb) [file 428_2017_2288_MOESM1_ESM.docx]

Supplement Table 1
