# Supplementary material for: Multicenter validation of cancer gene panel-based next-generation sequencing for translational research and molecular diagnostics
Source: Virchows Arch. 2018 Jan 27;472(4):557–65. doi: 10.1007/s00428-017-2288-7 (PMC5924673; doi:10.1007/s00428-017-2288-7)
Supplement: Supplementary file 2 — Variant allelic frequencies (%) of analyzed tumor DNAs derived from all sequencing sites (PGM™: a, b, and c; MiSeq™: d and e) that applied commercial broad cancer panels (CHPv2 or TSACP) for NGS. Samples not analyzed are indicated by “X”. Variants not detected are indicated by open circles “○”. WT = wild type. Local or central DNA extraction is indicated. (DOCX 257 kb) [file 428_2017_2288_MOESM2_ESM.docx]

Supplement Table 2
