# Supplementary material for: Multicenter validation of cancer gene panel-based next-generation sequencing for translational research and molecular diagnostics
Source: Virchows Arch. 2018 Jan 27;472(4):557–65. doi: 10.1007/s00428-017-2288-7 (PMC5924673; doi:10.1007/s00428-017-2288-7)
Supplement: Supplementary file 3 — Detected variant allelic frequencies (%), using centrally extracted tumor DNAs and cancer-specific gene panels at all sequencing sites (PGM™: a, b, c; MiSeq™: d, e, f, and g). Mean value (AVG) and standard deviation (STDN) are given. WT = wild type. (DOCX 266 kb) [file 428_2017_2288_MOESM3_ESM.docx]

Supplement Table 3
