# Supplementary material for: Multicenter validation of cancer gene panel-based next-generation sequencing for translational research and molecular diagnostics
Source: Virchows Arch. 2018 Jan 27;472(4):557–65. doi: 10.1007/s00428-017-2288-7 (PMC5924673; doi:10.1007/s00428-017-2288-7)
Supplement: Supplementary file 4 — Additional variants consistently detected at all sequencing sites using the commercial cancer gene panel-based NGS approach. Allelic variant frequencies are indicated (%). Sequencing site “c” did not perform local DNA extractions. (DOCX 266 kb) [file 428_2017_2288_MOESM4_ESM.docx]

Supplement Table 4
