# Supplementary material for: Multicenter validation of cancer gene panel-based next-generation sequencing for translational research and molecular diagnostics
Source: Virchows Arch. 2018 Jan 27;472(4):557–65. doi: 10.1007/s00428-017-2288-7 (PMC5924673; doi:10.1007/s00428-017-2288-7)
Supplement: Supplementary file 5 — Methods used for local DNA extraction of FFPE patient samples at four sequencing sites. DNA concentrations (ng/μl) and total volume (μl) of individual samples are shown. Samples in which known mutations have not been detected by targeted NGS are highlighted (filled gray). (DOCX 46 kb) [file 428_2017_2288_MOESM5_ESM.docx]

Supplement Table 5
